# Inhibiting proliferation of gefitinib-resistant, non-small cell lung cancer

**DOI:** 10.1007/s00280-013-2132-y

**Published:** 2013-03-21

**Authors:** Makoto Sudo, Tan Min Chin, Seiichi Mori, Ngan B. Doan, Jonathan W. Said, Makoto Akashi, H. Phillip Koeffler

**Affiliations:** 1 Cancer Science Institute, National University of Singapore, Singapore, Singapore; 2 Department of Hematology and Oncology, National University Hospital, Singapore, Singapore; 3 Department of Cancer Genomics, The Cancer Institute, Japanese Foundation for Cancer Research, Tokyo, Japan; 4 Department of Pathology and Laboratory Medicine, University of California, Los Angeles, USA; 5 Research Center for Radiation Emergency, National Institute of Radiological Sciences, Chiba, Japan; 6 Division of Hematology and Oncology, Cedars-Sinai Medical Center, Los Angeles, USA

**Keywords:** Non-small cell lung cancer (NSCLC), Tyrosine kinase inhibitor (TKI), EGFR, 17-DMAG, Belinostat, Combination chemotherapy

## Abstract

**Purpose:**

Sensitivity to a tyrosine kinase inhibitor (TKI) is correlated with the presence of somatic mutations that affect the kinase domain of epidermal growth factor receptor (EGFR). Development of resistance to TKI is a major therapeutic problem in non-small cell lung cancer (NSCLC). Aim of this study is to identify agents that can overcome TKI resistance in NSCLC.

**Methods:**

We used a carefully selected panel of 12 NSCLC cell lines to address this clinical problem. Initially, the cell lines were treated with a variety of 10 compounds. Cellular proliferation was measured via MTT assay. We then focused on the gefitinib-resistant, EGFR mutant cell lines [H1650: exon 19 and PTEN mutations; and H1975: exons 20 (T790M) and 21 (L858R)] to identify agents that could overcome TKI resistance.

**Results:**

Both 17-DMAG (Hsp90 inhibitor) and belinostat (histone deacetylase inhibitor, HDACi) effectively decreased the growth of almost all NSCLC lines. Also, belinostat markedly decreased the expression of EGFR and phospho-Akt in the cells. Combination of 17-DMAG and belinostat synergistically inhibited in vitro proliferation of these cells. Furthermore, both agents and their combination almost completely prevented TKI-resistant tumor formation (EGFR T790M mutation) in a xenograft model.

**Conclusion:**

These results suggest that the combination of 17-DMAG and belinostat should be examined in a clinical trial for TKI-resistant NSCLC cell.

**Electronic supplementary material:**

The online version of this article (doi:10.1007/s00280-013-2132-y) contains supplementary material, which is available to authorized users.

## Introduction

Lung cancer is the leading cause of cancer-related deaths, accounting for one-third of worldwide deaths from cancer. Non-small cell lung cancer (NSCLC) represents 80 % of lung cancers [1, 2]. The epidermal growth factor (EGFR) is an important therapeutic target in NSCLC [3]. Gefitinib was the first small-molecule EGFR tyrosine kinase inhibitor (TKI) that received FDA approval in 2003 [4]. Initial clinical studies found that only a minority of individuals with NSCLC responded to either gefitinib or erlotinib [5]. Asian non-smoking females were noted to be particularly sensitive to this class of drugs [4, 6]. Soon, investigators discovered that sensitivity to these TKIs was correlated with somatic mutations of the kinase domain of EGFR, such as either deletions within exon 19 or a L858R mutation in exon 21 [3, 7–9]. Individuals with these tumors often responded to TKI therapy, but usually had progressive disease after 6–12 months of therapy. These resistant tumors often acquired either an additional mutation (T790M) in exon 20 of EGFR or a second mutation in the downstream pathway of EGFR, both resulting in the development of resistance to EGFR-TKI [10–12].

A major therapeutic priority is to discover drugs and/or drug combinations that can inhibit proliferation of NSCLCs after they have developed an EGFR mutation that renders these cells TKI-resistant. After examining 10 different potential therapeutic compounds, we focused on two of the most active: 17-DMAG (Hsp90 inhibitor) and belinostat (HDACi). The Hsp90 chaperone complex is necessary for stability and activity of client proteins required for cellular homeostasis [13], and inhibition of Hsp90 results in proteasome degradation of its client proteins [14]. HDACi acetylates both histones and thousands of nonhistone cellular proteins confounding our understanding of how they mediate their anticancer affect. 17-DMAG and belinostat have different modes of action and toxicities. We found that both compounds potently suppressed proliferation of NSCLC cells, and when combined, they markedly inhibited these cells both in vitro and in vivo. Our data show that this drug combination overcomes TKI resistance in NSCLC cells.

## Materials and methods

### Reagents and NSCLC cell lines

The following compounds were used in this study: LY-333531 [ALEXIS Biochemicals (CA, USA)]; cisplatin [APP Pharmaceuticals, LLC (IL, USA)]; panobinostat [Selleck Chemicals (TX, USA)]; gefitinib; paclitaxel, docetaxel, gemcitabine and dasatinib [LC Laboratories (MA, USA)]; and 17-DMAG and belinostat [provided by National Cancer Institute (USA)]. All reagents were dissolved in dimethyl sulfoxide (DMSO), and the final concentration of DMSO never exceeded 0.1 % in culture. Antibodies, Akt (pan), phospho-Akt (Ser473), p70S6K, phospho-p70S6K (Thr421/Ser424), EGF receptor, phospho-EGF receptor (Tyr1068) and GAPDH were purchased from Cell Signaling (MA, USA).

The human NSCLC cell lines: H1299, H1666, H460, H520, PC9, HCC827, H1650 and H1975 were purchased from the American Type Culture Collection (Manassas, VA). HCC2279, HCC2935, HCC4006 and H820 were kindly provided by Dr. Roman K. Thomas (Max Planck Institute for Neurological Research). All cell lines were cultured in RPMI1640 supplemented with 10 % fetal calf serum, 100 U/ml of penicillin, and 100 μg/ml of streptomycin. The H1975 and H820 cell lines had EGFR T790M mutations in exon 20, associated with gefitinib and erlotinib resistance.

### Cell proliferation assays

NSCLC cells were seeded at a density of 4,000–15,000 per well in 96-well plates. After 24 h, cells were cultured with multiple concentrations of drugs for 72 h. Cell proliferation was determined by using CellTiter 96^®^ Non-Radioactive Cell Proliferation Assay (Promega) according to the manufacturer’s instructions, and the plates were read by a fluorescence spectrometer. Half-maximal inhibitory concentrations (“IC_50_-values”) were determined from the images under the growth inhibition curves using Prism 4.0 software.

### Western blot analysis

Cells were harvested, washed with PBS, and lysed in ProteoJET Mammalian Cell Lysis Regent (Fermentas) supplemented with ProteoBlock Protease Inhibitor Cocktail (Fermentas). Protein concentrations were measured by Bradford assay (Bio-Rad, Richmond, CA). Equal amounts of protein were dissolved in sodium dodecyl sulfate–polyacrylamide gel electrophoresis sample loading buffer and electrophoresed in a polyacrylamide gel (7.5 or 10 %). After electrophoresis, the proteins were electrotransferred to a polyvinylidene difluoride membrane (Immobilon, Millipore, Bedford, MA). Immunoblotting was performed using each antibody and detected by ECL-Plus reagent (Amersham, Boston, MA).

### In vivo experiments

Murine experiments were done in accord with a protocol reviewed and approved by the Institutional Animal Care and Usage Committee at the University of Singapore, in compliance with the Guide for the Care and Use of Laboratory Animals. The TKI-resistant (EGFR exons 20 and 21 mutations) H1975 cells (1 × 10^7^ cells) in the presence of matrigel (BD Biosciences) were injected into the flanks of nude mice (Biological Resource Center Singapore) (day 1). Each treatment group consisted of two tumors on the flanks of seven to eight mice. Doses of drugs (day 2–day 30) were as follows: belinostat (50 mg/kg); 17-DMAG (15 mg/kg); and combination therapy (belinostat 50 mg/kg and 17-DMAG 15 mg/kg). After day 30, dose of drugs was decreased: belinostat (25 mg/kg); 17-DMAG (7.5 mg/kg); and combination therapy (belinostat 25 mg/kg and 17-DMAG 7.5 mg/kg). The drugs were given intraperitoneally (i.p.) every other day. Control mice received i.p. injections with the diluent l-arginine (100 mg/kg) in water. Every tumor was measured with a caliper every other day, and the volume was calculated using the formula: (long diameter) × (short diameter)^2^/2. At the end of experiments, blood chemistries were measured (ALP, CRE, GLU, PHOS, CA, BUN, AST, ALT, TBIL, TP, AMYL, ALB, Na, K, Cl and LDL). The mice were anesthetized, and their tumors were carefully dissected, weighted, and divided into half. Half was used for immunochemistry, and the other was used for real-time RT-PCR and Western blotting.

### Immunohistochemistry

Paraffin-embedded tumor sections (4 μm) were placed on glass slides. This was followed by deparaffinization, rehydration, blocking of endogenous peroxidase activity and heat-induced antigen retrieval. The slides were stained with murine monoclonal Ki67 [clone MIB1(dilution 1: 100), DakoCytomation (CA, USA)]. The signal was detected using the MACH 2 Mouse HRP Polymer [Biocare Medical (CA, USA)]. All sections were visualized with the diaminobenzidine reaction and counterstained with hematoxylin.

### Statistical analysis

Data were collected using a minimum of three experiments and used to calculate the mean ± SD. Statistical significance was calculated using ANOVA or Bonferroni/Dunn multiple comparison test was considered significant at *p* values <0.05.

## Results

### Growth inhibition of gefitinib-resistant NSCLC

A panel of NSCLC cell lines were selected to encompass three groups: cohort #1, no mutation of EGFR (H1666, H460, H1299 and H520); cohort #2, EGFR mutation of either exon 19 or 21, and an additional TKI resistance-inducing mutation (H1650, H1975 and H820); and cohort #3, EGFR mutation of exon 19 and sensitive to TKI inhibitors (PC9, HCC827, HCC2279, HCC2935 and HCC4006) [Table 1]. The cellular proliferation of this panel of 12 NSCLC cell lines was examined by MTT over 72 h in the presence of increasing concentrations of a variety of compounds (gefitinib, dasatinib, panobinostat, belinostat, 17-DMAG LY-333531, cisplatin, gemcitabine, paclitaxel and docetaxel). Dose–response curves were generated, and the concentration that caused 50 % growth inhibition (IC_50_) was calculated (Table 1). Almost all cell lines were highly sensitive (IC_50_ range 0.037–0.29 μM) to dasatinib (src inhibitor) except the EGFR “wild-type” cell lines H1666 and H460 (IC_50_ range >10 μM). All NSCLC cell lines were highly sensitive (IC_50_ range 0.0006–0.14 μM) to panobinostat (HDACi), and almost all NSCLC cell lines were sensitive (IC_50_ range 0.29–1.2 μM) to belinostat (HDACi) except the EGFR-normal H1666 cell line (IC_50_ range >10 μM). 17-DMAG (Hsp90 inhibitor) (IC_50_ range 0.0005–0.095 μM) and paclitaxel and docetaxel (mitotic inhibitors) (IC_50_ range 0.0014–0.029 μM) had prominent antiproliferative activity. LY-333531 (PKCβ inhibitor) required a high concentration (IC_50_ range 4.7 to >10 μM) to inhibit cell growth. H1650 and H820 cells (EGFR mutant, TKI-resistant) were more resistant (IC_50_ 0.65, 1.2 μM, respectively) to gemcitabine (nucleoside analog) than the EGFR “wild-type” cell lines (IC_50_ range 0.17–0.4 μM). Antiproliferative activity of cisplatin (platinum-based chemotherapy) was also correlated with their EGFR gene mutational status (*p* = 0.0486) (EGFR mutant, TKI-sensitive > EGFR wild-type > EGFR mutant, TKI-resistant) (Supplement Fig. 1).

**Table 1:** Inhibition of proliferation of NSCLC cells were seeded in 96-well plates

Inhibition of proliferation of NSCLC cells [IC_50_ (μM)]												
	EGFR wild-type				EGFR mutant, TKI-resistant			EGFR mutant, TKI-sensitive				
	H1666	H460	H1299	H520	H1975	H1650	H820	PC9	HCC2279	HCC827	HCC2935	HCC4006
Gefitinib	>10	>10	>10	>10	8.2	>10	4.5	0.002	0.008	0.004	0.04	0.23
Dasatinib	>10	>10	0.07	0.27	0.12	0.18	0.29	0.11	0.037	0.06	0.17	0.4
Panobinostat	0.12	0.05	0.04	0.016	0.03	0.04	0.005	0.016	0.015	0.009	0.029	0.06
Belinostat	>10	0.86	1.2	0.75	0.68	0.88	0.4	0.29	0.4	0.29	0.97	0.46
17-DMAG	0.06	0.005	0.04	0.06	0.02	0.008	0.02	0.005	0.095	0.03	0.0005	0.003
LY-333531	>10	>10	>10	>10	>10	>10	>10	>10	4.7	6.2	4.7	7.9
Cisplatin	3.2	8	5.7	3	4.6	1.6	2.3	5	>10	5.2	>10	5.1
Gemcitabine	0.4	0.35	0.2	0.17	0.097	0.65	1.2	0.36	0.096	0.33	0.074	0.071
Paclitaxel	0.013	0.004	0.03	0.02	0.004	0.004	0.012	0.029	0.016	0.007	0.006	0.004
Docetaxel	0.004	0.003	0.008	0.004	0.002	0.004	0.005	0.006	0.005	0.003	0.0014	0.003

After 24 h, cells were cultured with multiple concentrations of drugs for 72 h. Cell proliferation was determined by using CellTiter 96^®^ non-radioactive cell proliferation assay. Half-maximal inhibitory concentrations (“IC_50_-values” μM) were determined

### Inhibition of EGFR expression

**Fig. 1:**
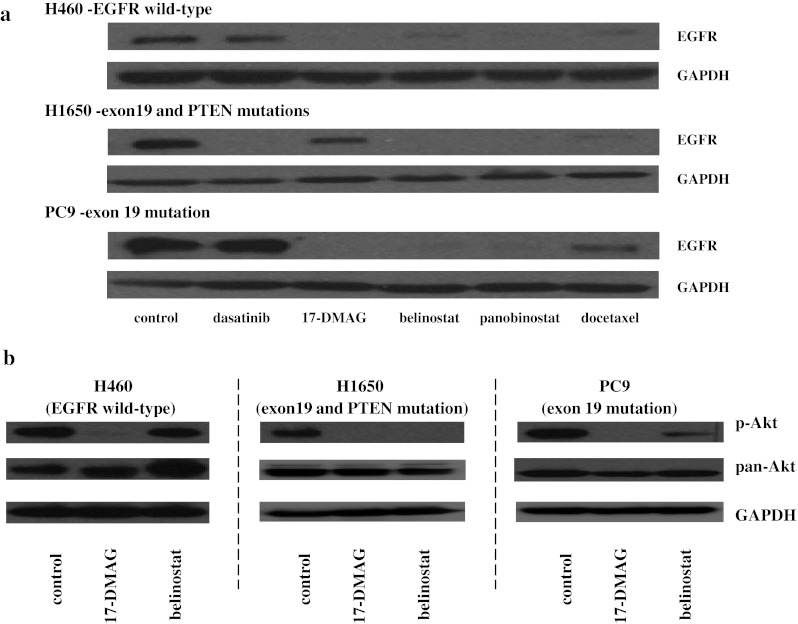
17-DMAG and belinostat decreased EGFR expression in all subtypes of NSCLC. **a** H460 (TKI-resistant, EGFR wild-type), H1650 (TKI-resistant, EGFR exon 19 and PTEN mutations) and PC9 (TKI-sensitive, EGFR exon 19 mutation) cells were treated for 24 h with either dasatinib (200 nM), 17-DMAG (50 nM), belinostat (500 nM), panobinostat (50 nM) or docetaxel (10 nM). Lysates were Western blotted and probed with antibody against EGFR and GAPDH (loading control). **b** 17-DMAG and belinostat decrease H460, H1650 and PC9 cells, which were treated for 24 h with 17-DMAG (50 nM) and belinostat (500 nM), Western blotted and probed with antibody against p-Akt (Ser473), pan-Akt and GAPDH (loading control)

A representative NSCLC cell line of each group [“wild-type” (H460 cells), TKI-resistant (exon 19 and PTEN mutant, H1650 cells) and TKI-sensitive (exon 19 mutant, PC9 cells)] was selected for further study. Mutant EGFR is a major driver of cell proliferation. 17-DMAG, belinostat, panobinostat and docetaxel decreased the levels of EGFR in cells from all three NSCLC groups (Fig. 1a). 17-DMAG, belinostat and panobinostat also decreased the levels of EGFR in H1975 cells (EGFR exons 20 and 21 mutations and TKI-resistant) (Supplement Fig. 2A). Dasatinib did not decrease expression of EGFR in the cells with either wild-type EGFR or exon 19 mutant, PC9 (Fig. 1a).

### Inhibition of activated Akt (p-Akt) downstream of EGFR

**Fig. 2:**
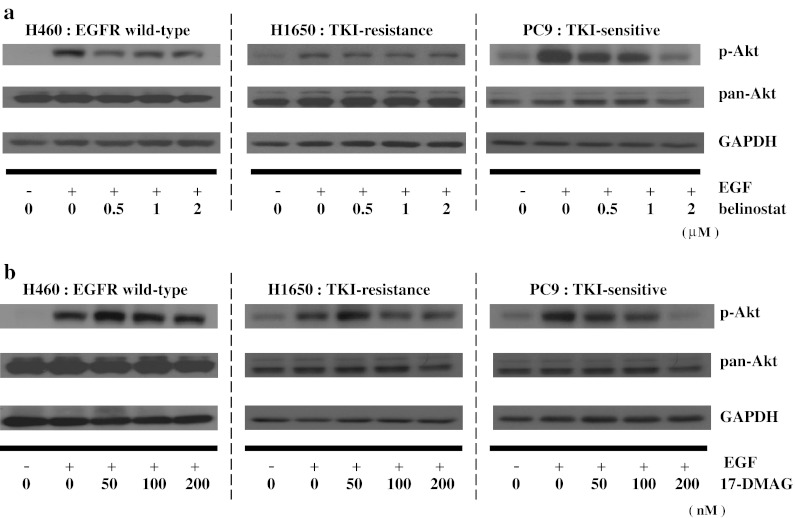
Levels of p-Akt in the three subtypes of NSCLC. Cells were serum-starved (16 h), treated with either belinostat [0, 0.5, 1, 2 μM] (**a**) or 17-DMAG [0, 50, 100, 200 nM] (**b**) for 4 h, stimulated with EGF (10 ng/ml, 15 min), cell lysate harvested and examined for levels of p-Akt (Ser473), pan-Akt and GAPDH (loading control) by Western blotting

Activated Akt (p-Akt) is one of the important molecules in the activated EGFR signaling pathway. 17-DMAG attenuated the expression of p-Akt in all three subgroups of cell lines (Fig. 1b). Belinostat decreased levels of p-Akt in the TKI-resistant [H1650 and H1975 (Supplement Fig. 2B)] and TKI-sensitive (PC9) EGFR mutant cell lines. In contrast, expression of total Akt either decreased only modestly or even increased in the three subtypes of NSCLC after exposure to each of the compounds (Fig. 1b). We also examined the ability of these two agents to inhibit p-Akt after serum starvation and short exposure (4 h) to EGF (Fig. 2). This model system tests the ability of the compound to inhibit a major growth stimulation pathway after exposure to activating ligand. Belinostat decreased p-Akt in H460 (EGFR wild-type) and PC9 (TKI-sensitive), but not in H1650 cells (TKI-resistant); however, starting levels of p-Akt in these cells were extremely low after EGF stimulation alone (Fig. 2a). 17-DMAG did not modulate levels of p-Akt in H460 (EGFR wild-type), but did decrease expression in the PC9 cells (TKI-sensitive) and the H1650 cells (TKI-resistant) (Fig. 2b).

### Combination of 17-DMAG and belinostat markedly inhibits cell proliferation of NSCLC in vitro

**Fig. 3:**
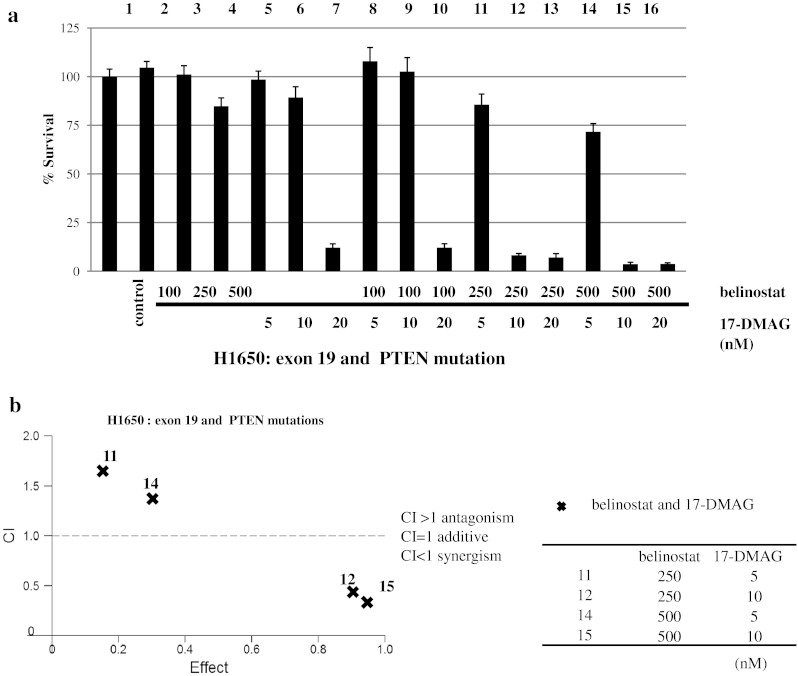
Combination of 17-DMAG and belinostat markedly inhibited cell proliferation of NSCLC. Co-treatment with belinostat (100, 250 and 500 nM) and 17-DMAG (5, 10 and 20 nM) for 72 h inhibited growth of H1650 cells (TKI-resistant, EGFR exon 19 and PTEN mutations) greater than either inhibitor alone (**a**). Calcusyn software (Biosoft) was used to analyze **a** data. CI < 1, CI = 1 and CI > 1 represent synergism, additive and antagonism of the two compounds, respectively (**b**)

**Fig. 4:**
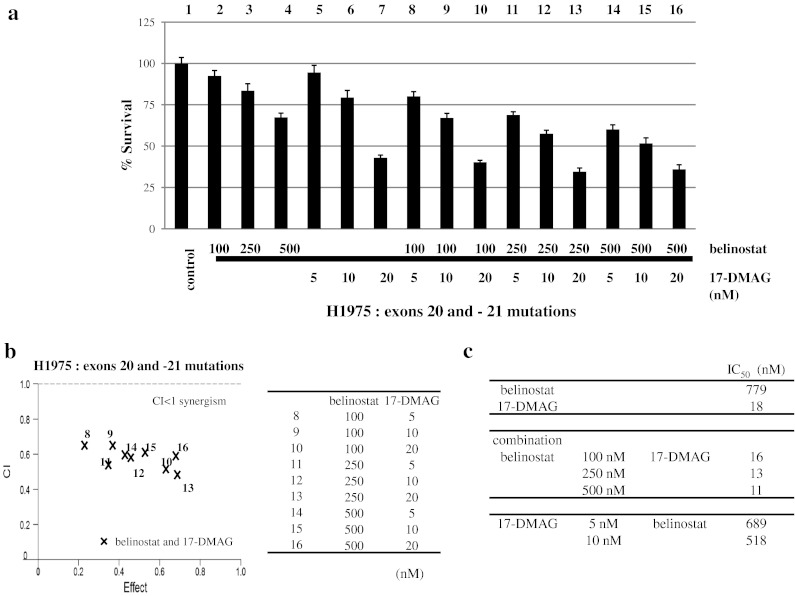
Combination of 17-DMAG and belinostat synergistically inhibited cell proliferation of TKI-resistant cells. Co-treatment with belinostat (100, 250 and 500 nM) and 17-DMAG (5, 10 and 20 nM) for 72 h inhibited growth of H1975 cells (TKI-resistant, EGFR exons 20 and 21 mutations) greater than either inhibitor alone (**a**). Calcusyn software (Biosoft) was used to analyze **a** data. CI < 1, CI = 1 and CI > 1 represent synergism, additive and antagonism of the two compounds, respectively (**b**). IC_50_ at each concentration of combination of belinostat and 17-DMAG was determined (**c**)

The above data suggested that both 17-DMAG and belinostat can inhibit growth of gefitinib-resistant NSCLC cells. Therefore, we examined whether the combination of 17-DMAG and belinostat versus either alone had an enhanced antiproliferative activity in H1650 cells. Co-treatment with 17-DMAG (5, 10 or 20 nM) and belinostat (100, 250 or 500 nM) inhibited growth of H1650 NSCLC cells (EGFR exon 19 and PTEN mutations), greater than either inhibitor alone, with 10 nM of 17-DMAG and either 250 or 500 nM of belinostat showing synergistic antiproliferative activity (Fig. 3a, columns 12 and 15, Fig. 3b). Likewise, the combination of 17-DMAG and belinostat synergistically inhibited the growth of the H1975 cells (TKI-resistant) (Fig. 4). The most effective combination was 250 nM of belinostat with 20 nM of 17-DMAG (Fig. 4a, b). In the presence of 250 nM of belinostat, IC_50_ of 17-DMAG was 13 nM (Fig. 4c).

### Combination of 17-DMAG and belinostat inhibits the in vivo growth of tumors derived from TKI-resistant human NSCLC cells

**Fig. 5:**
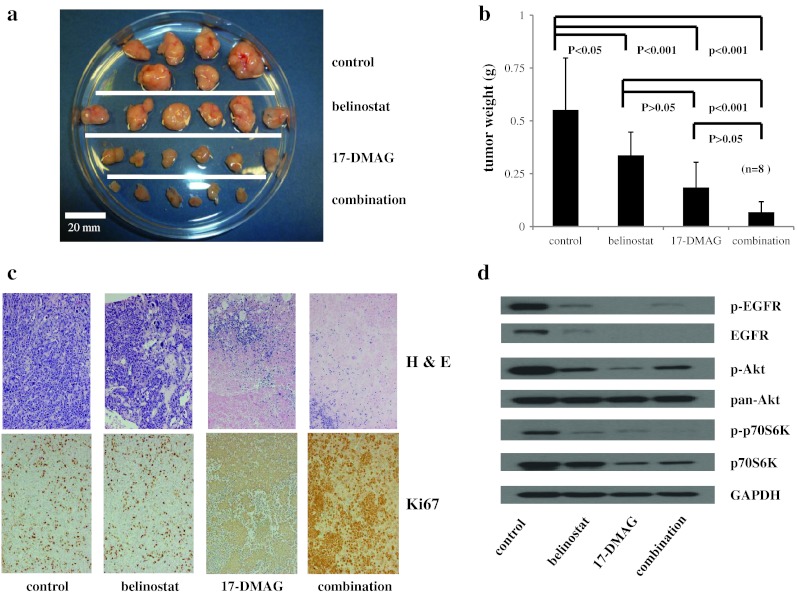
Combination of 17-DMAG and belinostat inhibited the in vivo growth of human TKI-resistant NSCLC xenografts. H1975 cells [TKI-resistant, EGFR exons 20 (T790M) and 21 mutations] were injected in the flanks of nude mice (day 1). On alternate days (day 2–30), mice received i.p. injections of either belinostat (50 mg/kg); 17-DMAG (15 mg/kg); combination therapy (belinostat 50 mg/kg and 17-DMAG 15 mg/kg) or diluent control. After day 30, dose of drugs was decreased by 50 % [belinostat (25 mg/kg); 17-DMAG (7.5 mg/kg); and combination therapy (belinostat 25 mg/kg and 17-DMAG 7.5 mg/kg)] and was continued every other day, limited day 54. Visual display of the dissected tumors from each cohort (**a**). Mean ± SD of the weights of the tumors was calculated. Statistical differences between the cohorts were determined using Bonferroni/Dunn multiple comparison test (**b**). Tumor sections were stained with hematoxylin and eosin, and immunohistochemistry for Ki67 was done (cell proliferation). Magnification was ×200 (**c**). Cell lysates were made from the dissected tumors and examined for levels of p-EGFR, EGFR, p-Akt, pan-Akt, p-p70S6K and p70S6K and GAPDH (loading control) by Western blotting (**d**)

Immunodeficient mice with xenografts of H1975 cells (EGFR exons 20 and 21 mutations and TKI-resistant) were treated with 15 mg/kg of 17-DMAG and/or 50 mg/kg of belinostat (every other day). Control mice received diluent alone. 17-DMAG, belinostat and their combination suppressed nearly completely the tumor growth with no significant difference in tumor size between each treatment group (data not shown). All of these mice had no apparent toxicity. In order to determine whether enhanced suppression tumor growth occured in the drug combination, we decreased by 50 % the dose of each compound on day 30 (7.5 mg/kg, 17-DMAG and/or 25 mg/kg, belinostat). Tumors of the Belinostat-treated mice increased more than those of the 17-DMAG-treated mice. Combination of 17-DMAG and belinostat was more effective than either drug alone. Examining for drug toxicities was analyzed on day 54 for 16 different blood chemistries; no significant differences occurred between control and experimental mice (data not shown). Likewise, the mice were weighed every other day, and no significant difference occurred in their weights. The mice were euthanized, and tumors were carefully dissected and weighed (Fig. 5a). Mean tumor weight of each individual treatment group was significantly less than that of the control cohort (*p* < 0.05, belinostat, *p* < 0.001, 17-DMAG). The mean weight of the tumors in the combination group was significantly less than that of either treatment cohort alone (*p* < 0.001) (Fig. 5a, b). 17-DMAG and the combination of belinostat and 17-DMAG treatment produced extensive necrosis with neutrophil infiltration (Fig. 5c). Protein was extracted from the tumors and Western blotted (Fig. 5d). Levels of p-EGFR, total EGFR, p-Akt, and p-p70S6K were decreased by each of the treatments compared to control. Total p70S6K was only decreased in tumors of mice that received either 17-DMAG or the combination of 17-DMAG and belinostat (Fig. 5d).

## Discussion

A major clinical problem often occurs for patients who have either an exon 19 or 21 mutation in their EGFR gene. These patients respond to gefitinib, but after a period of time, their tumors develop a T790M exon 20 mutation. These tumors no longer respond to the drug. We show here that tumors with T790M mutations are inhibited in their proliferation by belinostat or 17-DMAG. Furthermore, the combination of both of these compounds synergistically inhibits growth of these tumors.

The growth inhibitory effects of either belinostat or 17-DMAG were independent of the EGFR mutational status of the cells (*p* = 0.2323, *p* = 0.4455, respectively) (Supplement Fig. 1B, C); thus, their inhibitory activities were quite different from gefitinib. Effective cell kill by gefitinib was highly correlated with the presence of somatic mutations of EGFR (exons 19 and 21), affecting the kinase domain of EGFR (*p* < 0.0001) (Supplement Fig. 1A). In contrast, the “EGFR mutant, TKI-sensitive” cohort was more resistant to cisplatin than was the “wild-type” and the “EGFR mutant, TKI-resistant” cell lines [IC_50_ average 15.2, 5.0 and 2.83 (μM), respectively] (Supplement Fig. 1D).

Belinostat treatment decreased the levels of EGFR in all subtypes of NSCLC (Fig. 1a), resulting in downregulation of EGFR signaling pathway, thus blunting potential EGFR-mediated antiapoptotic and pro-survival signals. Belinostat (500 nM) treatment decreased within 24 h, the constitutive levels of phospho-Akt in H1650 cells (TKI-resistant, EGFR exon 19 and PTEN mutations) and PC9 cells (TKI-sensitive, EGFR exon 19 mutation). We previously showed that a HDAC inhibitor (vorinostat) profoundly decreased the levels of members of the activated Akt pathway in lymphoma cells, associated with their decreased proliferation [15]. The p-Akt in H460 cells (EGFR wild-type) was not decreased by belinostat (Fig. 1b). The lack of effect on constitutively activated p-Akt in these cells may be explained because H460 cells have a mutation of PI3K and KRAS [16]. Thus, belinostat may not be able to inhibit the ability of mutant PI3K and KRAS to activate Akt (Fig. 1b).

The mechanism by which HDAC inhibitors suppress oncogenic pathways is unclear. One hypothesis is that HDAC inhibitors activate “tumor suppressor genes” that subsequently result in growth inhibition, differentiation and apoptosis of cancer cells [17]. We previously combined a HDAC inhibitor (vorinostat) and a demethylating agent and noted the initiation of expression of a number of tumor suppressor genes in a variety of tumors including NSCLC, endometrial, prostate, ovary and pancreatic cancers [15, 18–22]. HDAC inhibitors also have the ability to regulate a number of key regulatory processes through acetylation of nonhistone proteins including p53, STAT, FOXO, HIF-1α, E2F1 and pRb [17, 23]. The HDAC inhibitor, belinostat, most likely suppresses several different oncogenic pathways, including the PI3K-Akt pathway as well as the activated EGFR (Fig. 1a, b).

The Hsp90 chaperone helps mediate the conformational maturation of several families of hormone receptors, transcription factors and kinases [24]. Inhibition of Hsp90 prevents its association with its client proteins, resulting in the conformational change of these target proteins causing an ubiquitin-mediated proteasomal degradation of the target proteins [25]. Cancer cells may be particularly dependent on chaperone proteins to survive a hypoxic, nutrient-starved microenvironment [26]. In addition, through the process of “oncogene addiction,” cancer cells may be dependent on either overexpressed or mutant kinases for viability, and hence making them particularly sensitive to Hsp90 inhibition. Mutant EGFR can mediate oncogenic addiction, and a Hsp90 inhibitor can prominently lower the levels of this addicting protein as shown by others [27] and ourselves here. We and others showed that 17-DMAG decreased the levels of EGFR in all subtypes of NSCLC, showing that this Hsp90 inhibitor modulates these receptors independent of the EGFR mutational status (Fig. 1a) [28]. Furthermore, Hsp90 stabilizes the active (phosphorylated) form of Akt since inhibition of binding of Akt-Hsp90 resulted in Akt dephosphorylation causing a loss of Akt kinase activity [24, 25, 29]. We found that 17-DMAG decreased the levels of activated Akt in all subtypes of NSCLC (Fig. 1b). Furthermore, when these cells were serum-starved and stimulated by EGF, 17-DMAG blunted the activation of p-Akt in H460 cells and PC9 cells (Fig. 2b). Therefore, the decrease in p-Akt in response to 17-DMAG may be related to both depletion of upstream EGFR, as well as direct effects on stability and phosphorylation of Akt. Early clinical trials have presented a mixed picture. Use of IPI-504, a water-soluble Hsp90 inhibitor monotherapy [randomized phase II trial] in advanced NSCLC, did not show clinical activity [30]. Another Hsp90 inhibitor, [ganetespib (STA-9090)], showed promising disease control in previously heavily pretreated NSCLC patients [phase II trial]. This drug appeared to be effective in all subsets of NSCLC including those with EGFR mutations, ALK translocations and KRAS mutations [31].

Combination of 17-DMAG and belinostat resulted in in vitro enhanced inhibition of proliferation of the TKI-resistant NSCLC cells (Figs. 3, 4). A previous study showed that panobinostat caused acetylation of Hsp90, reducing its association with mutant EGFR, Akt and STAT3, resulting in ubiquitination and depletion of these growth-promoting proteins in NSCLC cells [24, 25]. Therefore, our combination of a HDAC and a Hsp90 inhibitor may lead to a profound disruption of the chaperone function of Hsp90 with its client proteins, resulting in increased polyubiquitylation and proteasomal degradation of the client protein. In addition, we showed that either 17-DMAG or belinostat, or their combination inhibited TKI-resistant NSCLC xenografts growing in immunodeficient mice (Fig. 5a). Further, the combination of both therapies prominently decreased the mean tumor weight compared to tumor weights of those mice who received either belinostat or 17-DMAG alone (Fig. 5b). Similar to our in vitro results, Western blotting of cell lysates of tumors showed that either belinostat or 17-DMAG decreased expression of EGFR, p-Akt and its downstream signaling molecules (Fig. 5c). These mice had no chemical or physical manifestations of toxicity.

In summary, our data show that belinostat and 17-DMAG markedly inhibit the proliferation of the TKI-resistant NSCLC cells associated with decreased levels of both EGFR and activated Akt (p-Akt). Combination therapy with these compounds has the potential to be a therapeutic strategy for patients with EGFR-TKI-resistant NSCLCs.

## Electronic supplementary material

Below is the link to the electronic supplementary material.

**Supplement Fig. 1.** Relationship of EGFR status and sensitivity to gefitinib, belinostat, 17-DMAG, and cisplatin. Comparison of growth inhibition of EGFR “wild-type” cell lines (H1666, H460, H1299 and H520) versus TKI-resistant cell lines [H1650 (EGFR exon 19 and PTEN mutations, H1975 [EGFR exon 20 (T790M) and -21 mutations], H820 (EGFR exon 19 and -20 (T790M) mutations)] versus TKI-sensitive cell lines [mutation of EGFR exon 19: (HCC2279, HCC2935, PC9 and HCC4006)] to gefitinib [A], belinostat [B], 17-DMAG [C], and cisplatin [D]. Using the IC_50_ of NSCLC cell data [Table 1], one-way ANOVA was performed using Prism 4.0 software. (PDF 61 kb)

**Supplement Fig. 2.** 17-DMAG and belinostat decreased EGFR expression in TKI-resistant cells. 17-DMAG and belinostat decreased EGFR expression in TKI-resistant cells. H1975 cells [TKI-resistant, EGFR exon 20 (T790M) and -21 mutations] were treated for 24 h with either dasatinib (200 nM), 17-DMAG (50 nM), belinostat (500 nM), panobinostat (50 nM) or docetaxel (10 nM). Lysates were made, western blotted and probed with antibody against EGFR and GAPDH (loading control) (A). 17-DMAG and belinostat decreased levels of activated Akt (p-Akt) in TKI-resistant cells. H1975 cells (T790M) were treated with 17-DMAG (50 nM) and belinostat (500 nM) for 24 h. Cells were harvested, lysates made, subjected to Western blotting and probed with antibody against p-Akt (Ser473), pan-Akt and GAPDH (loading control) (B). (PDF 16 kb)
