# Supplementary figures and images for: Inhibiting proliferation of gefitinib-resistant, non-small cell lung cancer
Source: Cancer Chemother Pharmacol. 2013 Mar 21;71(5):1325–34. doi: 10.1007/s00280-013-2132-y (PMC3636434; doi:10.1007/s00280-013-2132-y)

Supplement Fig. 1

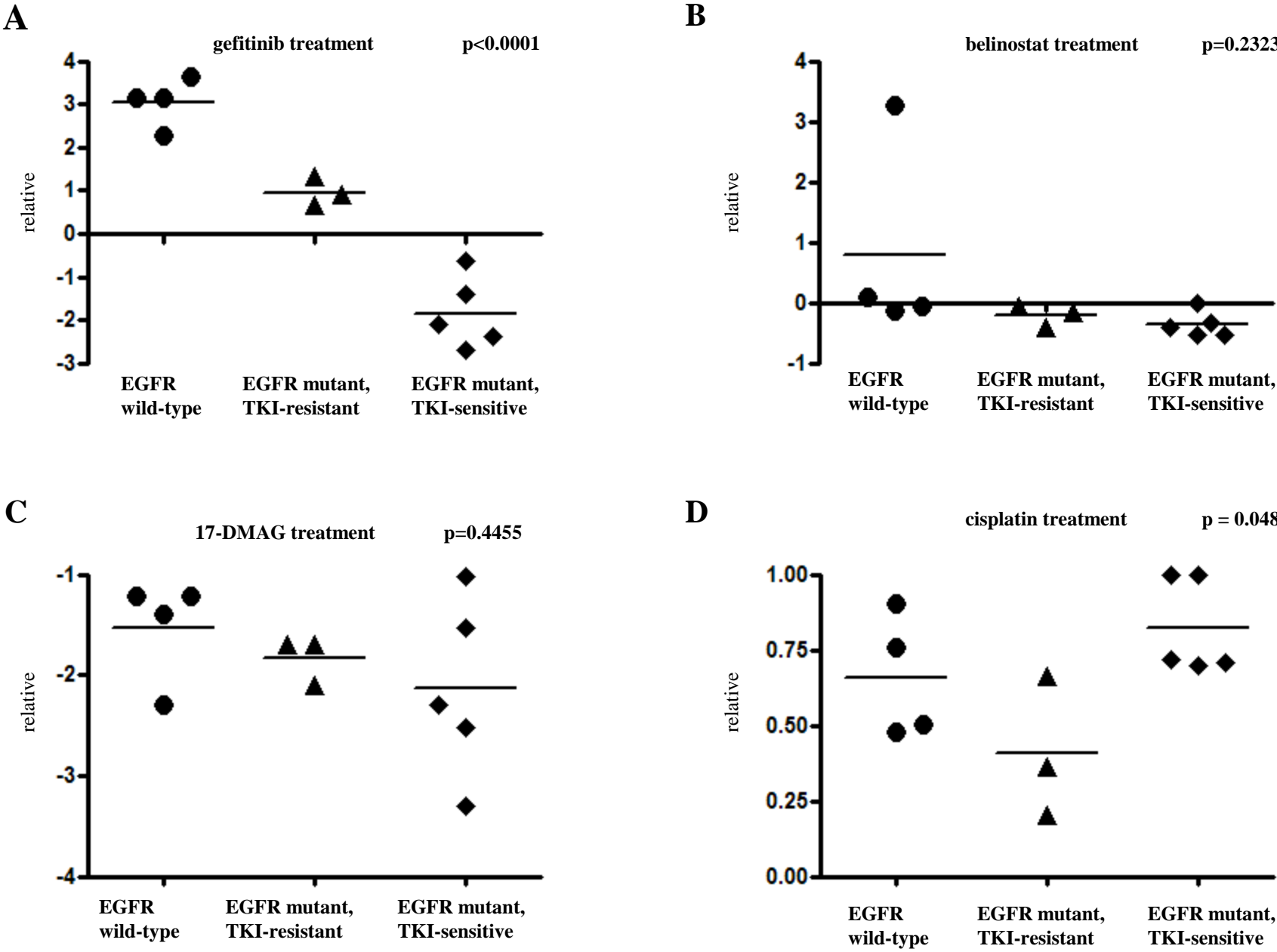

Supplement: Supplementary file 1 — Supplement Fig. 1. Relationship of EGFR status and sensitivity to gefitinib, belinostat, 17-DMAG, and cisplatin. Comparison of growth inhibition of EGFR “wild-type” cell lines (H1666, H460, H1299 and H520) versus TKI-resistant cell lines [H1650 (EGFR exon 19 and PTEN mutations, H1975 [EGFR exon 20 (T790M) and -21 mutations], H820 (EGFR exon 19 and -20 (T790M) mutations)] versus TKI-sensitive cell lines [mutation of EGFR exon 19: (HCC2279, HCC2935, PC9 and HCC4006)] to gefitinib [A], belinostat [B], 17-DMAG [C], and cisplatin [D]. Using the IC50 of NSCLC cell data [Table 1], one-way ANOVA was performed using Prism 4.0 software. (PDF 61 kb) [file 280_2013_2132_MOESM1_ESM.pdf]

**Supplement Fig. 2.**

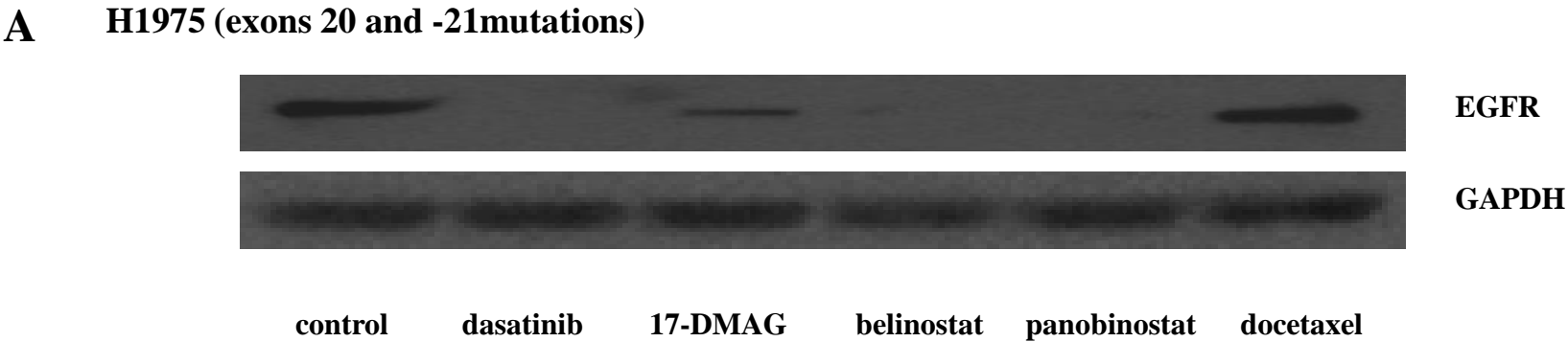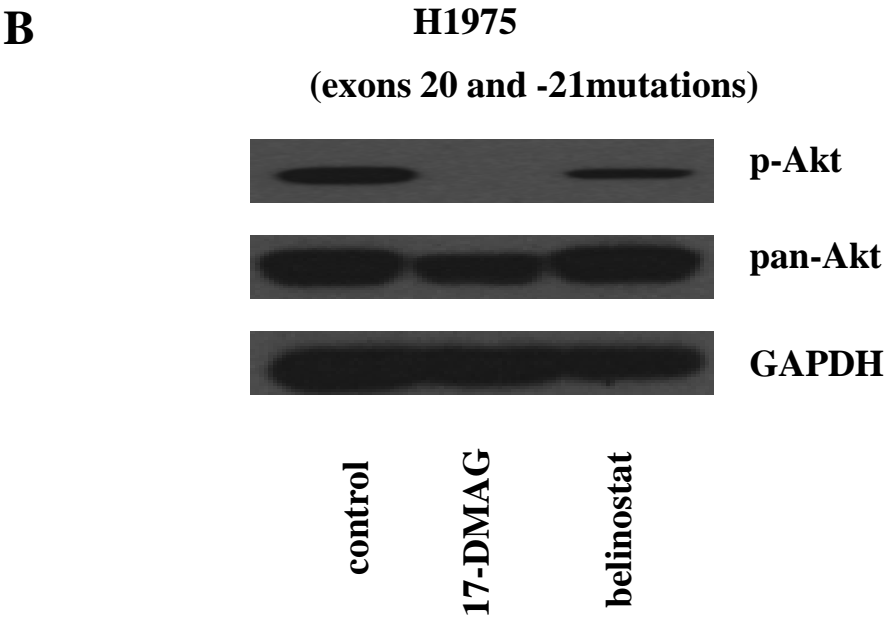

Supplement: Supplementary file 2 — Supplement Fig. 2. 17-DMAG and belinostat decreased EGFR expression in TKI-resistant cells. 17-DMAG and belinostat decreased EGFR expression in TKI-resistant cells. H1975 cells [TKI-resistant, EGFR exon 20 (T790M) and -21 mutations] were treated for 24 h with either dasatinib (200 nM), 17-DMAG (50 nM), belinostat (500 nM), panobinostat (50 nM) or docetaxel (10 nM). Lysates were made, western blotted and probed with antibody against EGFR and GAPDH (loading control) (A). 17-DMAG and belinostat decreased levels of activated Akt (p-Akt) in TKI-resistant cells. H1975 cells (T790M) were treated with 17-DMAG (50 nM) and belinostat (500 nM) for 24 h. Cells were harvested, lysates made, subjected to Western blotting and probed with antibody against p-Akt (Ser473), pan-Akt and GAPDH (loading control) (B). (PDF 16 kb) [file 280_2013_2132_MOESM2_ESM.pdf]
